# Endochin-like quinolones (ELQs) and bumped kinase inhibitors (BKIs): Synergistic and additive effects of combined treatments against *Neospora caninum* infection *in vitro* and *in vivo*

**DOI:** 10.1016/j.ijpddr.2021.08.007

**Published:** 2021-08-28

**Authors:** Nicoleta Anghel, Dennis Imhof, Pablo Winzer, Vreni Balmer, Jessica Ramseier, Kai Haenggeli, Ryan Choi, Matthew A. Hulverson, Grant R. Whitman, Samuel L.M. Arnold, Kayode K. Ojo, Wesley C. Van Voorhis, J. Stone Doggett, Luis M. Ortega-Mora, Andrew Hemphill

**Affiliations:** a Institute of Parasitology, Vetsuisse Faculty, University of Bern, Switzerland; b Graduate School for Cellular and Biomedical Sciences (GCB), University of Bern, Switzerland; c Center for Emerging and Re-emerging Infectious Diseases (CERID), Division of Allergy and Infectious Diseases, Department of Medicine, University of Washington School of Medicine, Seattle, WA, USA; d Department of Pharmaceutics, University of Washington, Seattle, WA, USA; e Departments of Global Health and Microbiology, University of Washington, Seattle, WA, USA; f VA Portland Health Care System, Research and Development Service, Portland, OR, USA; g SALUVET, Animal Health Department, Faculty of Veterinary Sciences, Complutense University of Madrid, Ciudad Universitaria s/n, Madrid, Spain

**Keywords:** Endochin-like quinolones, Bumped kinase inhibitors, Cytochrome *bc*_*1*_, Calcium-dependent protein kinase inhibitor, Neosporosis, Tachyzoites, Antiparasitic therapy, Combination therapy

## Abstract

The apicomplexan parasite *Neospora caninum* is an important causative agent of congenital neosporosis, resulting in abortion, birth of weak offspring and neuromuscular disorders in cattle, sheep, and many other species. Among several compound classes that are currently being developed, two have been reported to limit the effects of congenital neosporosis: (i) bumped kinase inhibitors (BKIs) target calcium dependent protein kinase 1 (CDPK1), an enzyme that is encoded by an apicoplast-derived gene and found only in apicomplexans and plants. CDPK1 is essential for host cell invasion and egress; (ii) endochin-like quinolones (ELQs) are inhibitors of the cytochrome *bc*_*1*_ complex of the mitochondrial electron transport chain and thus inhibit oxidative phosphorylation. We here report on the *in vitro* and *in vivo* activities of BKI-1748, and of ELQ-316 and its respective prodrugs ELQ-334 and ELQ-422, applied either as single-compounds or ELQ-BKI-combinations. *In vitro*, BKI-1748 and ELQ-316, as well as BKI-1748 and ELQ-334, acted synergistically, while this was not observed for the BKI-1748/ELQ-422 combination treatment. In a *N. caninum*-infected pregnant BALB/c mouse model, the synergistic effects observed *in vitro* were not entirely reproduced, but 100% postnatal survival and 100% inhibition of vertical transmission was noted in the group treated with the BKI-1748/ELQ-334 combination. In addition, the combined drug applications resulted in lower neonatal mortality compared to treatments with single drugs.

## Highlights

• BKI-1748 is a bumped kinase inhibitor with anti-Neospora activity *in vitro* and *in vivo*
• Endochin-like quinolones target the cytochrome *bc*1 complex and exhibit anti-Neospora properties.
• Single and combined applications of these two compound classes do not interfere in pregnancy outcome in mice
• BKI-1748/ELQ-334 combination treatment results in 100% postnatal survival and blocks vertical transmission in the neosporosis mouse model

## Graphical abstract

**Figure:**
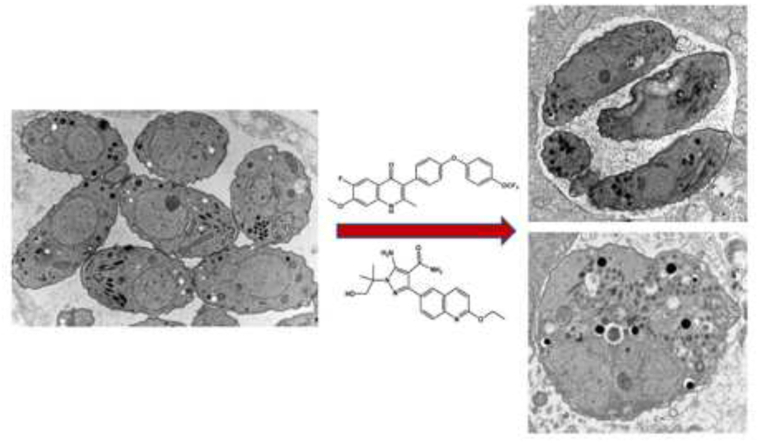

## 1 Introduction

*Neospora caninum* is an obligatory intracellular coccidian parasite, closely related to *Toxoplasma gondii* (Dubey et al., 2017), and was first described in 1988 by Dubey et al. (1988). There are three infective stages which comprise an elaborate life cycle: (i) the sporozoites encapsulated in oocysts, which are formed in the intestine of the definitive hosts (Dubey et al., 2017) (dogs (McAllister et al., 1998), grey wolves (Dubey et al., 2011), dingoes (King et al., 2010) and coyotes (Gondim et al., 2004)), and are shed through faeces in the environment; (ii) the tachyzoites, which represent the proliferative and disease-causing stage; and (iii) the bradyzoites, which proliferate slowly and form intracellular tissue cysts that can persist for extended periods of time without causing inflammatory reactions. Infection can take place orally, through ingestion of bradyzoite-infected tissue cysts or accidently by uptake of oocysts. However, the most important route of *N. caninum* infection is vertical (transplacental) transmission from the dam to the foetus by tachyzoites (Dubey et al., 2017). Despite the wide range of intermediate hosts, there is no evidence of infection in humans, thus in contrast to toxoplasmosis, neosporosis is not considered a zoonosis (McCann et al., 2008).

Most notably in the cattle industry, *N. caninum* causes serious economic constraints, with annual financial losses of more than one billion US dollars worldwide. This is due to abortion, stillbirth, and birth of weak and/or persistently infected calves, which then transmit the disease to the next generation (Reichel et al., 2013). Infection with *N. caninum* has no serious implications in an immunocompetent animal, but abortion can occur upon primary infection during pregnancy due to exogenous transplacental transmission, or upon recrudescence of a chronic infection during pregnancy (endogenous transplacental transmission) (Dubey et al., 2017; Horcajo et al., 2016; Monney and Hemphill, 2014). Despite the considerable economic impact of neosporosis worldwide, there is no commercial vaccine or drug on the market to either prevent or treat neosporosis infections in cattle (Aguado-Martínez et al., 2017).

Endochin-like quinolones (ELQs) are drugs that have proven activity against a wide range of apicomplexan parasites including *T. gondii*, *Plasmodium falciparum*, *Babesia microti* (Doggett et al., 2020) and *N. caninum* (Anghel et al., 2018). ELQs inhibit the cytochrome *bc*_*1*_ complex of the mitochondrial electron transport chain. Cytochrome *bc*_*1*_ facilitates the electron transfer from ubiquinol to cytochrome *c*, and contains an oxidative (*Q*_*o*_) and reductive (*Q*_*i*_) catalytic site (Stickles et al., 2015). One of the most efficacious ELQs for *T. gondii* and *N. caninum* is ELQ-316, that selectively acts on apicomplexan rather than human cytochrome *b* (Doggett et al, 2012, 2020). Moreover, ELQ-316 has been shown to confer *Q*_*i*_ site inhibition (Doggett et al., 2012)_._ The excellent *in vitro* activity of ELQ-316 could not be translated *in vivo* due to the poor solubility of this compound, leading to limited absorption and bioavailability when applied orally at higher doses (Doggett et al., 2020; Miley et al., 2015; Nilsen et al., 2013). To overcome this difficulty, prodrugs were synthesized, such as ELQ-334 and ELQ-422, which contain a carbonate ester pro-moiety that disrupts crystalline formation. These prodrugs are metabolized shortly after oral administration and subsequent release of the active compound, leading to improved absorption and efficacy (Doggett et al., 2020; Miley et al., 2015; Winter et al., 2011).

Bumped kinase inhibitors (BKIs) are another class of promising compounds which target the calcium-dependent protein kinase 1 (CDPK1), which is expressed in apicomplexan parasites, plants, and fungi, but not in mammalian cells. In apicomplexans, CDPK1 is involved in gliding motility, invasion, and egress of the invasive stages (Kieschnick et al., 2001; Winzer et al., 2015). BKIs compete with ATP for the kinase active site (Van Voorhis et al., 2017). The specificity of BKIs is mediated by scaffolds that are derived from either an adenosine-like pyrazolopyrimidine (PP) or a 5-aminopyrazole-4-carboxamide (AC) scaffold, that bind to the hinge region of the active site of CDPK1, similar to the binding motif of ATP, but with an attached bulky aromatic R1-substituent at the C3 position, representing the “bump” of the BKI (Keyloun et al., 2014). While the active site is accessible in apicomplexan CDPK1 that has a glycine gatekeeper residue, the structural feature of BKIs impairs binding to the active sites of almost all mammalian kinases, since they possess a bulkier gatekeeper residue (Van Voorhis et al., 2017). BKIs were reported to be active against *T. gondii* (Rutaganira et al., 2017) and *N. caninum* (Doggett et al., 2014; Müller et al., 2017b; Sánchez-Sánchez et al., 2019; Winzer et al., 2015, 2020b)*.* For the two PP compounds BKI-1294 (Ojo et al., 2014) and BKI-1553 (Müller et al., 2017a; Sánchez-Sánchez et al., 2018), proof of concept was demonstrated in small and large animal models of toxoplasmosis and neosporosis. In addition, BKIs efficacy have been reported against *Cryptosporidium parvum* (Castellanos-Gonzalez et al., 2013), *Babesia bovis* (Pedroni et al., 2016), *Sarcocystis neurona* (Ojo et al., 2016)*, Besnoitia besnoiti* (Jiménez-Meléndez et al., 2017), and *Cystoisospora suis* (Shrestha et al., 2019).

The search for novel treatment strategies against parasitic diseases includes the use of combination therapies, which have been developed for numerous diseases (Kappagoda et al., 2011; Rosenthal, 2003; Stickles et al., 2016). The use of combinations of drugs that target different metabolic pathways or distinct sites in a specific parasite can produce synergistic killing effects, and possibly reduced amounts that are required from the individual compounds (Sun et al., 2016). Combination therapy can also reduce drug resistance by hitting multiple targets at once, increase patient compliance due to potentially reduced treatment durations (Pink et al., 2005), and might lead to a new indication for the respective treatments (Sun et al., 2016). In this study, we assessed the efficacy of ELQ-316 and its two prodrugs ELQ-334 and ELQ-422, and BKI-1748, either individually or combinations thereof, to study potential synergistic and/or additive effects against *N. caninum* infection *in vitro* and in a pregnant neosporosis mouse model.

## 2 Materials and methods

### 2.1 Tissue culture media, biochemicals, and compounds

If not otherwise stated, cell culture media were purchased from Gibco-BRL (Zürich, Switzerland), and biochemical reagents were acquired from Sigma (St. Louis, MO, USA). The kits for molecular biology were purchased from Qiagen (Hilden, Germany). ELQs were synthesized as previously described (McConnell et al., 2018), and BKI-1748 was originally synthesized in the Department of Biochemistry of the University of Washington, USA (PMID 30423128 & PMID 30830766) and scaled up by WuXi Apptec Inc., Wuhan, China to >98% purity by LC-MS/MS and NMR. Compounds were shipped as powder. For *in vitro* studies, they were stored as dimethyl sulfoxide (DMSO) stock solutions at concentrations of 40, 20 or 10 mM at −20 °C. For *in vivo* experiments, BKI-1748, ELQ-334, ELQ-422 or combinations thereof were suspended in corn oil, followed by administration to mice by oral gavage.

### 2.2 Cell culture and parasites

Human foreskin fibroblasts (ATCC® PCS-201-010™) and BALB/c dermal fibroblasts (CELLNTEC AG, Bern, Switzerland) were cultured and maintained as described previously (Müller et al., 2016; Winzer et al., 2015, 2020b). The *N. caninum* Spain-7 strain (NcSp-7), and *N. caninum* strain expressing β-galactosidase gene (Nc-β-gal) were cultured in HFF cells and maintained and prepared for infections as described previously (Aguado-Martinez et al., 2016; Müller et al., 2016; Winzer et al., 2015).

### 2.3 Cytotoxicity of ELQs and BKI-1748

The cytotoxicity values of ELQ-316, ELQ-334 and BKI-1748 in HFF monolayers have been reported previously (Anghel et al, 2018, 2020). The cytotoxic effect of ELQ-422 was assessed by Alamar blue assay as previously described (Barna et al., 2013; Schorer et al., 2012). In short, non-infected HFF were grown to confluency in 96-well plates, followed by treatment with 200 μL medium containing either 20, 10, 5, 2.5, 1.3, 0.6, 0.3, 0.2, 0.08, 0.04, 0.02 and 0.01 μM of ELQ-422, respectively. Non-treated HFF and DMSO controls were included as controls. Plates were further cultured at 37 °C/5% CO_2_ for 72 h. After the medium was discarded by aspiration, cells were washed once with PBS. The resazurin stock solution was diluted 1:200 in PBS, and 200 μl were added into each well. Plates were read at *λ* excitation 530 nm and *λ* emission 590 nM at the Enspire multilabel reader (Perkin Elmer, Waltham). Fluorescence was measured at different timepoints. Relative fluorescence units were calculated from timepoints with linear increase.

### 2.4 In vitro proliferation measurements in cultures treated with BKI-1748 and ELQs individually or in combination

*In vitro* efficacy assessments of ELQ-316, ELQ-334, ELQ-422, BKI-1748, and respective drug combinations were done using Nc-β-gal (Barna et al., 2013; Schorer et al., 2012). Briefly, HFF monolayers were cultured in 96-well plates and were infected with 10^3^ Nc-β-gal tachyzoites in the presence of ELQ-316, ELQ-334, ELQ-422, and BKI-1748, as well as the combinations of BKI-1748 with each of the three ELQs. All drugs and combinations were prepared as serial dilutions of 20000, 2000, 200, 20, 2, 0.2, 0.02, 0.002 and 0.0002 nM in cell culture medium, respectively. The stock solutions of ELQs as well as the medium were heated up to 37 °C before use. After three days of culture (37 °C/5% CO_2_) in the presence of compounds, wells were washed with 200 μl PBS, and 90 μl of PBS containing 0.05% Triton X-100 were added into each well. After addition of 10 μl 5 mM chlorophenol red-β-D-galactopyranoside dissolved in PBS, the absorption shift was measured at a wavelength of 570 nM at various time points on an Enspire multilabel reader (Perkin Elmer, Waltham). The half-maximal effective concentrations (EC_50_ values) were calculated after the logit-log transformation of relative growth and subsequent regression analysis by use of the corresponding software tool contained in the Excel software package (Microsoft, Seattle, WA).

### 2.5 Immunofluorescence labelling of N. caninum tachyzoites treated with BKI-1748 and ELQ-422 individually or in combination

HFF monolayers were grown to 50% confluency on glass coverslips in 24-well plates and were infected with 3 × 10^4^ Nc-Sp7 tachyzoites. Three hours after infection, coverslips were treated either with 1 μM ELQ-422, 2.5 μM BKI-1748, or 1 μM ELQ-422 + 2.5 μM BKI-1748, and the cultures were further maintained in the presence of the drugs. Immunofluorescence staining was carried out as described (Imhof et al., 2021). Briefly, at different time points, the coverslips were washed once in PBS, fixed in 3% paraformaldehyde in PBS (pH 7.2) for 10 min, and permeabilized in a 1:1 solution of pre-cooled methanol/acetone at −20 °C (Alaeddine et al., 2013; Guionaud et al., 2010; Winzer et al., 2015). The primary antibodies were (i) anti-IMC1, a rabbit polyclonal antibody directed against the inner membrane complex (diluted 1:500), and (ii) a monoclonal mouse anti-SAG1 antibody directed against the major immunodominant *N. caninum* tachyzoite surface antigen 1 (diluted 1:1000) (Björkman and Hemphill, 1998). The corresponding secondary antibody conjugates were anti-mouse fluorescein isothiocyanate [FITC] and anti-rabbit tetramethyl-rhodamine-isothiocyanate (TRITC) conjugate (Sigma), both diluted at 1:300. After washing in PBS, coverslips were mounted in Vectashield mounting medium (Vector Laboratories, Burlingame, CA, USA) containing 4,6-diamidino-2-phenylindole (DAPI) (Anghel et al., 2018). All specimens were viewed on a Nikon Eclipse E800 digital confocal fluorescence microscope. Image processing was performed using the OpenLab 5.5.2 software (Improvision, PerkinElmer, Waltham, MA, USA).

### 2.6 Transmission electron microscopy (TEM)

TEM was carried out as described previously (Winzer et al., 2015). Briefly, 5 × 10^5^ HFF were grown in T25 flasks. Upon confluency, they were infected with 1 × 10^6^ NcSp-7 tachyzoites. After 4 h at 37 °C/5% CO_2_, the medium and non-invaded parasites were removed, flasks were washed twice with PBS and new medium was added. Subsequently, 1 μM ELQ-316, 1 μM ELQ-334 or 1 μM ELQ-422, and 2.5 μM of BKI-1748 were used as single-treatments, and combined treatments were done with 1 μM ELQ-316 + 2.5 μM BKI-1748 and 1 μM ELQ-422 + 2.5 μM BKI-1748. After further culture for 6, 12, 24, 48, or 72 h, the medium was discarded and the flasks were washed twice with 0.1 M Na-Cacodylate buffer (pH 7.3), followed by fixation in 2% glutaraldehyde in 0.1 M Na-Cacodylate buffer for 10 min at room temperature. Infected monolayers were carefully removed using a cell scraper, transferred into an Eppendorf tube and fixation was continued for 2 h. The specimens were post-fixed in 2% OsO_4_ in cacodylate buffer, washed in water, followed by immersion in UranyLess solution (Electron Microscopy Science, Hatfield PA, USA) for 30 min. Following stepwise dehydration in a graded series of ethanol, specimens were embedded in Epon-812 resin, with three changes of epoxy resin. Following polymerization at 60 °C, ultrathin sections were cut using an ultramicrotome (Reichert and Jung, Vienna, Austria), and were placed onto 200 mesh nickel grids (Plano GmbH, Marburg, Germany). Following staining with UranyLess and lead citrate, specimens were viewed on a PhilipsCM12 TEM operating at 80 kV.

### 2.7 Ethics statement

Protocols involving animals were approved by the Animal Welfare Committee of the Canton of Bern under the licenses BE101/17 and BE117/2020. Animals were handled in strict accordance with the practices made to minimize suffering. Female and male BALB/c mice were purchased at 6 weeks of age from a commercial breeder (Charles River, Sulzberg, Germany), and were maintained in a common room under controlled temperature with 14 h/10 h light and dark cycles, with food and water accessible *ad libitum*. All animals were housed in the animal facility for two weeks prior to experimentation for acclimatization, and procedures were carried according to the guidelines of the animal welfare legislation of the Swiss Veterinary Office.

### 2.8 Assessment of potential interference in pregnancy in BALB/c mice of ELQ-334, ELQ-422, and BKI-1748 as single compounds or combined

Thirty-six BALB/c female and 18 male mice, six weeks of age were used. Female mice were oestrus synchronized during three days by the Whitten effect (Whitten, 1957), and mated for three nights by housing one male with two females. After mating, males were removed and female mice were randomly allocated to six experimental groups (six mice per group, two females per cage). Compounds were administered in 100 μl corn oil on days nine to 13 of pregnancy calculated after the first day of mating, as follows: ELQ-334 was administered at 10 mg/kg, ELQ-422 at 10 mg/kg, and BKI-1748 was administered at 20 mg/kg. The combined treatment groups were as follows: ELQ-334 at 10 mg/kg + BKI-1748 at 20 mg/kg, and ELQ-422 at 10 mg/kg + BKI-1748 at 20 mg/kg. The placebo group received 100 μl corn oil for five consecutive days. One hour after the last administration of compound, blood was collected from the tail vein, and transferred to heparin-coated tubes, which were inverted carefully followed by centrifugation (10 min at 1000×*g*) and plasma was collected and stored at −20 °C for drug level analysis (Imhof et al., 2021). During the experimental period, mice were examined daily and weighed several times during pregnancy to determine the number of pregnant animals and to detect possible abortions (which would result in rapid weight loss). On day 18 post-mating, pregnant females were separated into individual cages, where they gave birth on days 20–22 post mating. Live and stillborn pups were counted, and dead pups removed. Data on the number of female mice that became pregnant, litter size (number of delivered pups per dam), stillborn mice (dead pups from birth until day two post-partum), post-natal mortality (dead pups from day three post-partum to the end of the experiment), and clinical signs of treated mice were recorded. Dams and live pups were monitored for two weeks after birth to rule out possible adverse effects due to treatments. At the end of the experiment, mice were euthanized in a euthanasia chamber using isoflurane and CO_2_.

### 2.9 Measurements of BKI-1748 and ELQ-316 concentration in BALB/c mouse plasma samples

BKI-1748 and ELQ-316 concentrations in mouse plasma samples were determined as previously described (Hulverson et al., 2019; Imhof et al., 2021). Briefly, BKI-1748 and ELQ-316 were extracted from plasma using acetonitrile/0.1% formic acid with an internal standard and a standard curve was prepared for quantification. All LC-MS/MS analytes were measured with an Acquity ultra performance liquid chromatography (UPLC) system in tandem with a Xevo TQ-S micro mass spectrometer (Waters, Milford, MA, USA).

### 2.10 ELQ-334, ELQ-422, and BKI-1748 treatments as single compounds or in combination in the pregnant neosporosis mouse model (experimental infection with NcSp-7 tachyzoites)

Ninety BALB/c female mice and 45 male mice, six weeks of age were used in this experiment. Oestrus synchronization and mating was performed as described above. After mating, (day 0) female mice were randomly assigned to seven groups: ELQ-334 (n = 14), ELQ-422 (n = 14), BKI-1748 (n = 14), ELQ-334 + BKI-1748 (n = 14), ELQ-422 + BKI-1748 (n = 14), positive control (corn oil treatment and infection, n = 14), and negative control (corn oil treatment without infection, n = 6). NcSp-7 tachyzoites were transferred from HFF cells to BALB/c dermal fibroblasts and maintained at 37 °C and 5% CO_2_ three days prior to infection (Imhof et al., 2021). On day 7, tachyzoites were harvested and prepared for infection as previously described (Arranz-Solís et al., 2015). In brief, tachyzoite-infected fibroblasts were recovered from culture flasks when they were >90% of intracellular tachyzoites still located in parasitophorous vacuoles, and infected cells were repeatedly passed through a 25-gauge needle at 4 °C. The number of viable tachyzoites was estimated by Trypan blue exclusion (typically 95–99%) followed by counting the viable tachyzoites in a Neubauer chamber. A sublethal dose of 10^5^ tachyzoites in 100 μl were administered to each mouse by subcutaneous injection. The negative control group was inoculated with 100 μl dermal fibroblasts. From two days post-infection (day 9), compounds were administered in 100 μl corn oil on days nine to 13 of pregnancy, calculated after the first day of mating, as follows: ELQ-334 was administered at 5 mg/kg, ELQ-422 at 7.5 mg/kg, and BKI-1748 was administered at 20 mg/kg. The combined treatment groups were: ELQ-334 at 5 mg/kg + BKI-1748 at 20 mg/kg, and ELQ-422 at 7.5 mg/kg + BKI-1748 at 20 mg/kg. The positive and negative controls received 100 μl corn oil for five consecutive days. On day 18, pregnant females were separated into individual cages, they gave birth on days 20–22, and were allowed to rear their pups for 28 days until day 48–50. Live and stillborn pups were counted, and dead pups were removed, and the non-pregnant mice were also monitored during the following 30 days until day 50. Dams and their offspring were evaluated for clinical signs of disease, and data on the number of dams, stillborn pups and those dying postnatally were monitored. All mice were euthanized four weeks post-partum as described above. Blood was collected by cardiac puncture, centrifuged, and serum was stored at −20 °C for subsequent analysis of IgG1 and IgG2a levels by ELISA. Brain was collected and stored at −20 °C prior to further processing.

### 2.11 Analysis of biological samples

For the quantification of the brain parasite load in all the mice, a real-time PCR method designed for quantification of *N. caninum* DNA was employed (Aguado-Martínez et al., 2019; Müller et al., 2002). DNA extraction and purification were done with the NucleoSpin DNA RapidLyse Kit (Macherey-Nagel, Oensingen, Switzerland) employing the standard protocols. DNA concentrations in all samples were determined using the QuantiFluor double-stranded DNA (dsDNA) system (Promega, Madison, WI, USA) according to the manufacturer's instructions and adjusted to 5 ng/μl with sterile DNase-free water. Quantitative real-time PCR was performed using the Light Cycler (Roche, Basel, Switzerland), and parasite loads calculated with a standard curve of *N. caninum* DNA samples of 10, 100, and 1000 tachyzoites included in each run.

Levels of IgG1 and IgG2a in *N. caninum* infected mice were assessed by ELISA as described previously (Debache et al, 2008, 2009). Briefly, 96-well plates were coated with 200 ng NcSp-7 soluble tachyzoite protein extract diluted in coating buffer (50 mM sodium bicarbonate; 50 mM sodium carbonate both dissolved in ultrapure water; pH 9.6) per well. After overnight incubation at 4 °C, plates were washed three times with washing buffer (0.05% PBS-Tween-20) before non-specific binding sites were blocked with blocking buffer (1% bovine serum albumin and 0.05% Tween-20 in PBS) for 2 h at room temperature. Four-fold serial dilution of each serum sample was performed in blocking solution including positive and negative control, before samples were applied and incubated for 90 min at room temperature. After three washes, secondary antibodies (goat anti-mouse-IgG1 or -IgG2a conjugated to alkaline phosphatase (SouthernBiotech, Birmingham, USA) diluted 1:5000 in blocking solution) were added to serum samples for 1 h incubation at room temperature. Alkaline phosphatase substrate was added to develop the enzymatic reaction and absorbance was measured as optical density (OD) at 405 nm in a microplate reader (EnSpire™ 2300 Multilabel Reader, PerkinElmer, Switzerland). The same positive and negative serum samples were used for each plate to be able to compare OD values between samples measured in different plates. OD values were converted into a relative index per cent (RIPC) value using the following formula [RIPC = (OD_405nm_ sample – OD_405nm_ negative control)/(OD_405nm_ positive control – OD_405nm_ negative control)*100] (Aguado-Martinez et al., 2016).

### 2.12 Statistics

Statistical analysis of cerebral parasite burdens and antibody titers between treatment groups were compared by the non-parametric Kruskal-Wallis test for the whole groups, followed by Mann-Whitney-U test. Pup mortality along time was compared by plotting survival events at each time point in Kaplan-Meier graphs and survival curves were compared by the Log-rank (Mantel-Cox) test. Chi-square test was performed for the neonatal and postnatal mortality. Statistical analyses were performed by using Graphpad Prism (GraphPad Software, La Jolla, CA, USA).

## 3 Results

### 3.1 In vitro effects of ELQs and BKI-1748, applied as single drugs or in combination

**Fig. 1:**
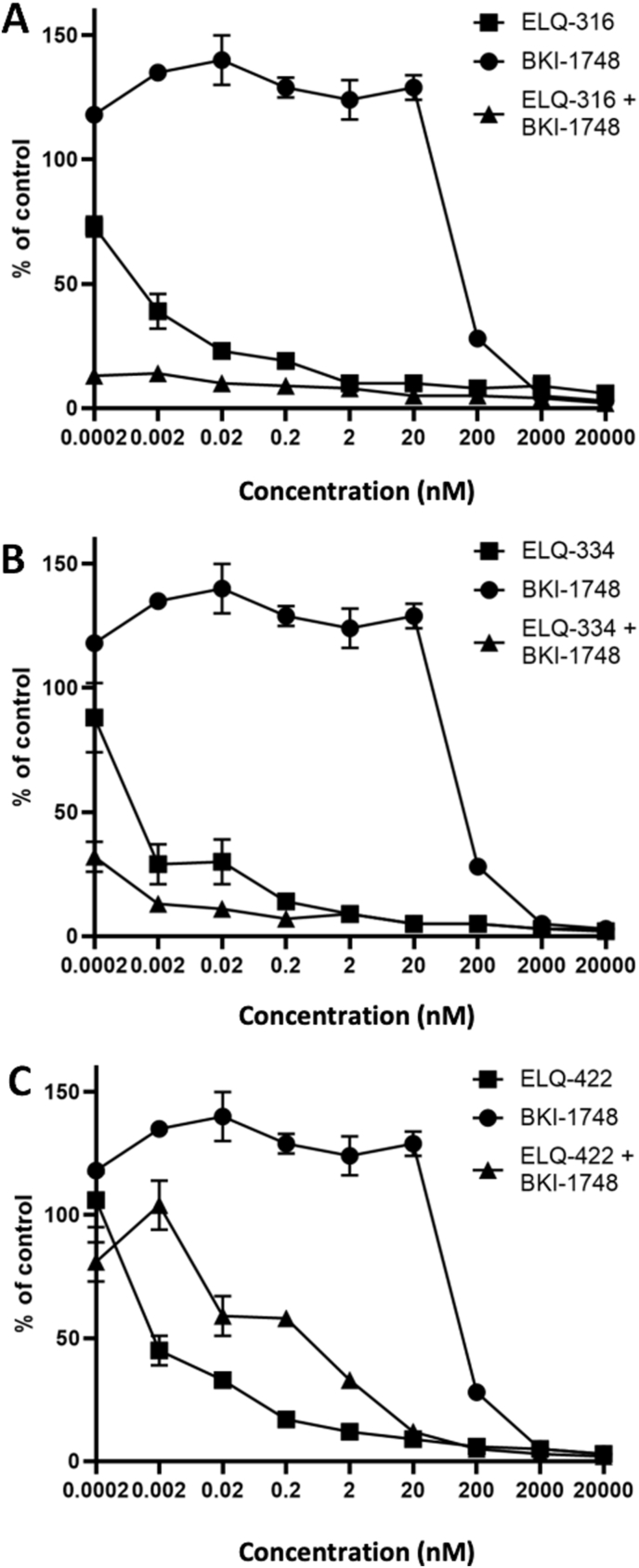
Relative growth curves of Nc-β-gal tachyzoites treated with ELQ-316, BKI-1748, and combined ELQ-316 + BKI-1748 (A), as well as ELQ-334 and its combination with BKI-1748 (B) and ELQ-422 and its combination with BKI-1748 (C) for 72 h, with compound added concomitantly to infection. A synergistic effect in terms of reduction of parasite proliferation can be observed in the case of ELQ-316 and ELQ-334 in combination with BKI-1748 (A and B), with inhibition curves of the combinations remaining at a lower level than the curves obtained with the single compounds.

To observe the inhibition of proliferation by ELQ-316, ELQ-334, ELQ-422, and BKI-1748 or respective combinations, the compounds were added concomitantly to infection of HFF monolayers by Nc-β-gal tachyzoites at different concentrations, and dose responses and EC_50_ values were calculated. Results are summarized in Table 1 and respective growth curves are shown in Fig. 1. ELQ-316 treatment resulted in an EC_50_ of 1.55 nM, while BKI-1748 had an EC_50_ of 165 nM. However, combination treatments with these two compounds resulted in a combined EC_50_ below 0.01 nM. ELQ-334, the ELQ-316 prodrug (EC_50_ = 54.2 nM) and BKI-1748 also acted synergistically with a combined EC_50_ below 0.1 nM. In the case of ELQ-422 (EC_50_ of 15.6 nM) the combination with BKI-1748 did not result in synergism, as the resulting combined EC_50_ of 53 nM was above the EC_50_ for ELQ 422, but well below the BKI-1748 EC_50_. Neither of the compounds impaired HFF viability at a concentration of 20 μM (Anghel et al, 2018, 2020) (data not shown).

**Table 1:** EC_50_ values of ELQs, BKI-1748 and their combination as determined by *N. caninum* transgenic strain expressing β-galactosidase.

Code	Structure	*N. caninum* EC_50_ (nM)
ELQ-316		1.55
ELQ-334		54.2
ELQ-422		15.6
BKI-1748		165^a^
ELQ-316 + BKI-1748	–	<0.01
ELQ-334 + BKI-1748	–	<0.1
ELQ-422 + BKI-1748	–	53

a Data from Imhof et al. (2021).

**Fig. 2:**
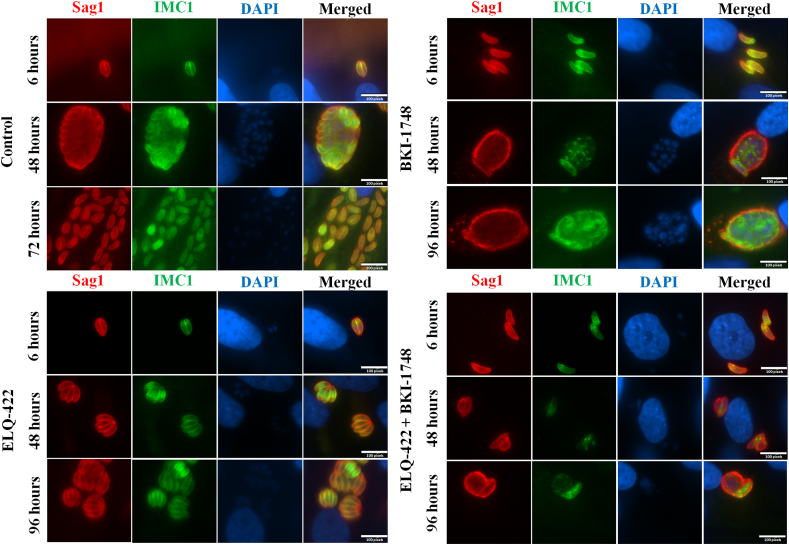
Immunofluorescence images of *N. caninum* tachyzoites grown in HFF treated with ELQ-422, BKI-1748 and the combination ELQ-422 + BKI-1748 for a period of 96 h, in comparison to untreated cultures. Red indicates the localization of the major tachyzoite antigen SAG1, green labels the inner membrane complex 1 (IMC1), which is a marker for newly formed zoites, blue indicates DNA stained by DAPI. In the control samples (A), lysis and re-infection of new host cells was observed latest at 72h post-infection. ELQ-422 treatment (B) slowed down tachyzoite proliferation, and treatment with BKI-1748 induced the formation of multinucleated complexes (C). The BKI-1748 + ELQ-422 combination treatment induced the formation of smaller complexes with a clearly diminished number of newly formed zoites (D). Scale bar = 9 μm. (For interpretation of the references to colour in this figure legend, the reader is referred to the Web version of this article.)

In order to observe the morphological changes induced by the combined ELQ-422 + BKI-1748, treatment, immunofluorescence labelling of treated cultures was performed (Fig. 2). NcSp-7 tachyzoites were allowed to invade HFF monolayer during 3 h prior to initiation of single or combination treatments. During the 72 h observation period, non-treated controls had proliferated extensively, formed large parasitophorous vacuoles after 48 h, and after 72 h many parasites had undergone egress and re-invaded neighbouring fibroblasts (Fig. 2 A). In contrast, in cultures treated with ELQ-422, proliferation of tachyzoites was markedly slowed down (Fig. 2 B), similar to what was observed previously with ELQ-334 and ELQ-316 (Anghel et al., 2018). In contrast, BKI-1748 treatment (Fig. 2 C) induced the formation of multinucleated complexes (MNCs), which were characterized by a marked SAG1 surface staining and remained intracellular. MNCs contained newly formed zoites as indicated by IMC1 labelling and were unable to complete cytokinesis and form tachyzoites. The combined ELQ-422 + BKI-1748 treatment (Fig. 2 D) resulted also in the formation of MNCs, but these complexes exhibited a reduced size compared to BKI-1748 treatment alone.

**Fig. 3:**
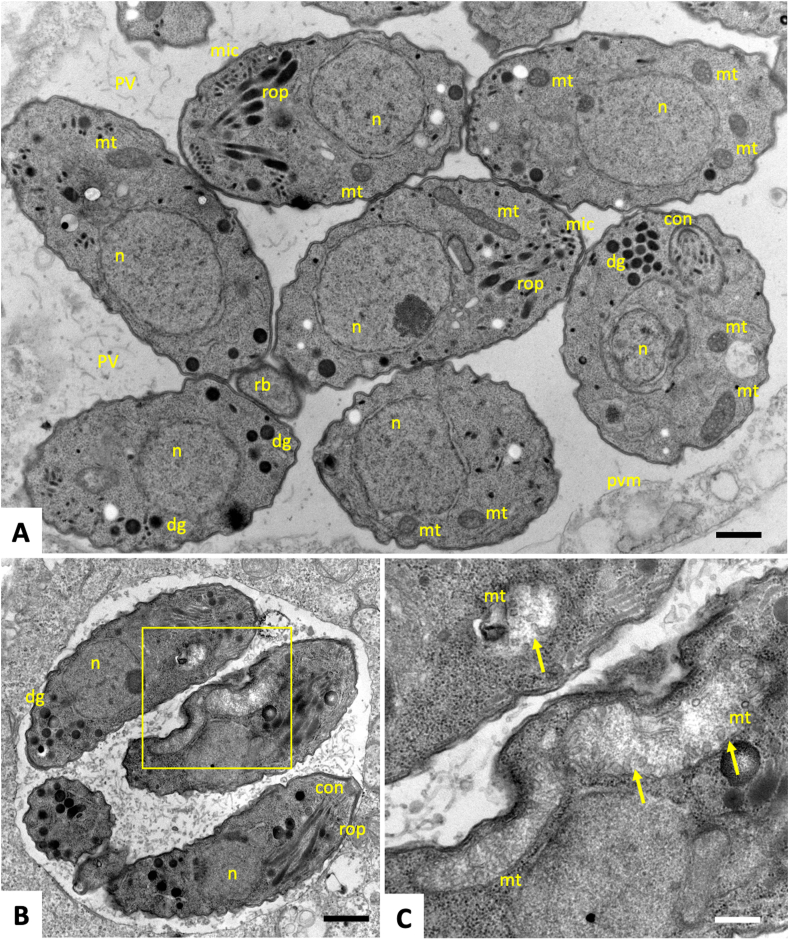
TEM of *N. caninum* tachyzoite-infected cultures. Cultures were left without treatment for 2 days (A) or were treated with ELQ-422 during 72 h (B, C). In non-treated cultures (A), tachyzoites formed a parasitophorous vacuole (PV), delineated by a parasitophorous vacuole membrane (pvm), containing numerous proliferating tachyzoites; rop = rhoptries, mic = micronemes, dg = dense granules, rb = residual body, n = nucleus, con = conoid, mit = mitochondrion. Similar features were found in ELQ-422 treated cultures, but the PV was distinctly smaller. The overall shape of tachyzoites remained intact, but the mitochondrial matrix was clearly distorted. The framed region in B is enlarged in C. Bars = 0.3 μm in A, 0.5 μm in B and 0.16 μm in C.

**Fig. 4:**
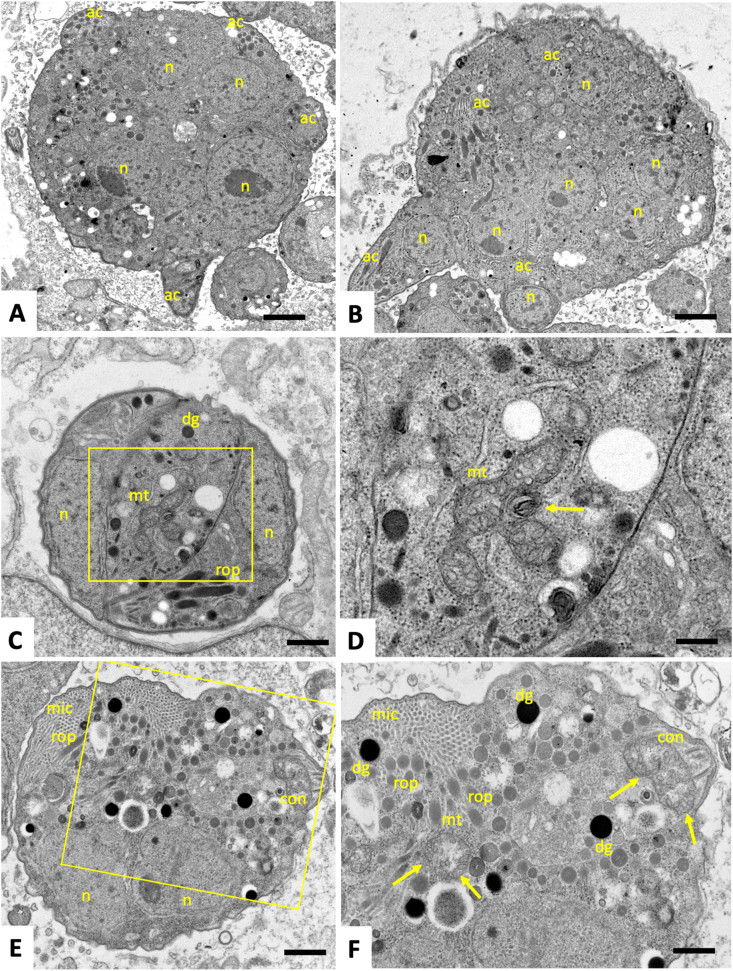
TEM of *N. caninum* infected cultures treated with BKI-1748 and the ELQ-422/BKI-1748 combination. (A) and (B) show parasites treated with BKI-1748 after 72 h, forming an intracellular multinucleated complex containing numerous nuclei (n), with small apical complexes (ac) emerging on the surface. Complexes in cultures treated with the combination (C–F) were of smaller size, contained a lower number of nuclei, but micronemes (mic), rhoptries (rop) and dense granules (dg) were clearly discernible, and the higher magnification views in (D) and (F) show distinct alterations in the mitochondrial matrix (indicated by arrows). Bars = 1 μm in A and B, 0.45 μm in C, 0.18 μm in D, 0.45 μm in E, 0.3 μm in F.

The ultrastructural effects of the ELQ-422 and/or BKI-1748 drug treatments were assessed by TEM. In non-treated cultures (Fig. 3 A), tachyzoites proliferating by endodyogeny were found to be localized within intracellular parasitophorous vacuoles, delineated by a parasitophorous vacuole membrane. Secretory organelles such as micronemes, rhoptries, and dense granules were clearly discernible, as well as the mitochondrion displaying an electron dense matrix. In contrast, treatment with ELQ-422 (Fig. 3 B, C) resulted in the formation of smaller parasitophorous vacuoles with a lower number of tachyzoites, which exhibited distinct alterations in the mitochondrion. These parasites lost the electron dense mitochondrial matrix, and the cristae were largely distorted. However, the overall shape and the integrity of the parasitophorous vacuole and its membrane remained intact, and parasites appeared still viable after 96 h of treatment. In contrast, exposure of *N. caninum-*infected HFF to BKI-1748 resulted in the formation of intracellular MNCs comprised of several nuclei and apical complexes of newly formed zoites (Fig. 4 A, B). ELQ-442 + BKI-1748 combination treatment, however, almost abolished proliferation, resulting in the formation of much smaller complexes with fewer nuclei, and mitochondria also appeared altered, with largely disorganized cristae structure, although these alterations appeared to occur to a lesser extent compared to ELQ-422-only treatment (Fig. 4C–F).

Thus, although a synergistic effect could not be seen based on proliferation measurement of Nc-β-gal tachyzoites, inspection by microscopical means demonstrated that combining ELQ-422 and BKI-1748 had an additive effect.

### 3.2 Safety and plasma levels after individual and combined drug application in pregnant BALB/c mice

*In vivo* studies were carried out with the ELQ-316 prodrugs ELQ-334 and ELQ-422. These two compounds exhibit increased absorption compared to ELQ-316, which in turn leads to increased exposure of ELQ-316 after metabolization of the compounds (Lawres et al., 2016). In the first experiment we assessed the potential interference of pregnancy in BALB/c mice upon oral application of ELQ-334, ELQ-422, BKI-1748, and the respective combinations, with ELQs applied at 10 mg/kg/day and BKI-1748 at 20 mg/kg/day, each five days in a row. The results are summarized in Table 2. Two of six mice became pregnant in the ELQ-344 and BKI-1748 groups, and four of six mice became pregnant in the ELQ-422 group. The combination treatment group between ELQ-334 and BKI-1748 had two out of six pregnant mice, and the group where ELQ-422 was combined with BKI-1748, four out of six mice became pregnant. These results indicate that none of the treatments had a dramatic impact on fertility. The treatment with 10 mg/kg ELQ-334 and 10 mg/kg ELQ-422 resulted in only little neonatal mortality (two out of 11 and one out of 17 pups, respectively). As previously reported, BKI-1748 applied at 20 mg/kg/day did not impair fertility rates or pup survival (Imhof et al., 2021). However, the combination treatments appeared to induce neonatal pup mortality to a higher degree (mortality of five out of 11 and three out of 19 pups for the combined ELQ-334 + BKI-1748 and ELQ-422 + BKI-1748 treatments, respectively), which raised some safety concerns. However, no postnatal mortality was observed in the ELQ-422, the ELQ-422 + BKI-1748, and the BKI-1748 groups.

**Table 2:** Results of pregnancy safety assessment in BALB/c mice of BKI-1748, the two ELQs, and the respective combinations. Drugs were formulated in corn oil and applied by gavage on days 9–13 of pregnancy at the indicated dosages.

Treatment	Fertility	Pups per dam	Neonatal mortality^a^	Postnatal mortality^b^
ELQ-334 10 mg/kg	2/6	11/2	2/11	1/9
ELQ-422 10 mg/kg	4/6	17/4	1/17	0/16
BKI-1748 20 mg/kg	2/6	11/2	0/11	0/11
ELQ-334 10 mg/kg + BKI-1748 20 mg/kg	2/6	11/2	5/11	1/6
ELQ-422 10 mg/kg + BKI-1748 20 mg/kg	4/6	19/4	3/19	0/16

a Pups that were born dead or died within 2 days post-partum.

b Pups that died during days 3–14 post-partum. Placebo control had no effect on pregnancy outcome, shown in previous results (Imhof et al., 2021).

**Fig. 5:**
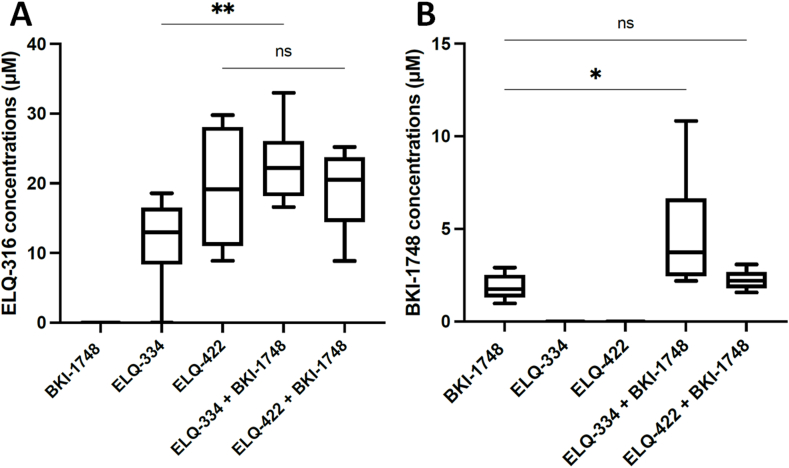
ELQ-316 (A) and BKI-1748 (B) plasma concentrations in BALB/c mice measured 1 h after the final treatments (day 5) with ELQ-334, ELQ-422, BKI-1748 and respective combination treatments. For each dosage, blood of six mice was retrieved through the tail vein and plasma was collected to measure the drug concentration by LC-MS/MS. ELQ-316 plasma concentrations (A) were significantly enhanced in the ELQ-334 + BKI-1748 combination treatment group compared to the group treated with ELQ-334 alone (**P < 0.0043), while combining ELQ-422 with BKI-1748 had no effect (ns = not significant). (B) The ELQ-334 + BKI-1748 combination treatment also resulted in significantly increased BKI-1748 plasma levels (*P < 0.0152), while combining BKI-1748 with ELQ-422 did not alter BKI-levels (ns = not significant). Statistical differences were performed using the non-parametric Kruskal-Wallis test followed by Mann Whitney-U test, included in the Graphpad prism software.

ELQ-316 and BKI-1748 levels were measured in plasma samples obtained from the mice of the five treatment groups 1 h after the last drug application (Fig. 5 and Table 3). In the ELQ-334 treated group, ELQ-316 plasma concentrations ranged between 11.16 and 18.58 μM, and in the combination therapy with BKI-1748, respective concentrations were significantly increased (16.60–32.99 μM; *P* < 0.0043). In the group treated with ELQ-422 at 10 mg/kg, the ELQ-316 concentrations were between 8.89 and 29.78 μM, and in the combination treatment with BKI-1748 at 20 mg/kg, the concentrations of ELQ-316 were between 8.86 and 25.21 μM, thus there was no difference between ELQ-422 administered alone or in combination with BKI-1748. For BKI-1748 administered alone at 20 mg/kg, the plasma concentrations reached levels between 0.98 and 2.91 μM. In the combination of BKI-1748 at 20 mg/kg and ELQ-334 at 10 mg/kg, concentrations of BKI-1748 were significantly increased from 2.20 up to 10.83 μM (*P* < 0.0152), and when combined with ELQ-422 at 10 mg/kg, the concentrations of BKI-1748 ranged between 1.57 and 3.08 μM, which was in a similar range as without ELQ-422. Overall, these plasma levels were clearly higher than the EC_50_s calculated for all the compounds which are in the nanomolar range, and especially the combination of ELQ-334 and BKI-1748 resulted in increased plasma levels for both drugs, while this was not evident for the ELQ-422 + BKI-1748 combination treatment.

**Table 3:** Plasma levels of ELQs and BKI-1748 measured after 5 days of drug treatments with individual compounds and respective combinations. Plasma samples were collected 1 h after the final drug application.

Treatment	Mouse P/NP^a^	Plasma concentration (μM)	
		ELQ-316	BKI-1748
ELQ-334 10 mg/kg	NP	N.D **	–
	P	12.71	–
	NP	11.16	–
	NP	15.81	–
	NP	13.24	–
	P	18.58	–
ELQ-422 10 mg/kg	P	8.89	–
	NP	11.73	–
	P	14.78	–
	P	27.49	–
	NP	23.49	–
	P	29.78	–
BKI-1748 20 mg/kg	NP	–	1.73
	NP	–	2.40
	P	–	0.98
	NP	–	1.78
	P	–	1.42
	NP	–	2.91
ELQ-334 10 mg/kg + BKI-1748 20 mg/kg	P	20.93	3.11
	NP	32.99	10.83
	P	23.76	4.37
	NP	23.52	2.20
	NP	16.60	2.54
	NP	18.70	5.24
ELQ-422 10 mg/kg + BKI-1748 20 mg/kg	NP	25.21	3.08
	P	8.86	1.88
	P	16.33	1.57
	NP	19.20	2.03
	P	21.82	2.53
	P	23.22	2.39

a P: pregnant mouse; NP: non-pregnant mouse. ** ND: Not done, insufficient plasma volume.

### 3.3 Efficacy of individual and combined drugs in pregnant BALB/c mice challenged by experimental N. caninum infection

In the challenge experiment, the dosages of the two ELQs were reduced due to previously shown tendency of these compounds to slightly interfere in pregnancy outcome. ELQ-334 was administered at 5 mg/kg, either as single treatment or also when applied in combination with BKI-1748. ELQ-422 was administered at 7.5 mg/kg alone, as well as in combination with BKI-1748. The dosage of BKI-1748 remained at 20 mg/kg.

**Fig. 6:**
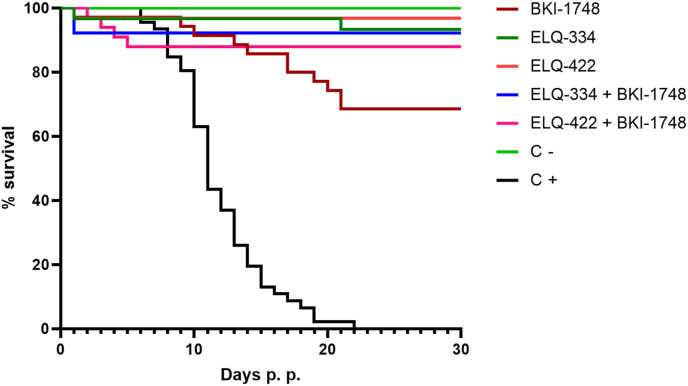
Kaplan-Meier survival curves of pups born from ELQ-334 (5 mg/kg), ELQ-334 (5 mg/kg) + BKI-1748 (20 mg/kg), ELQ-422 (7.5 mg/kg), ELQ-422 (7.5 mg/kg) + BKI-1748 (20 mg/kg) treated dams, and from positive (*N. caninum* infected and non-treated) and negative (non-infected, non-treated) control mice. Statistical differences between treated groups and positive control curves were highly significant (****P < 0.0001), calculated with the Log-rank (Mantel-cox) statistical test included in the Graphpad prism software.

In all the experimental groups, the fertility rates and litter sizes were not impacted by the treatment. All the treated groups influenced the pup survival compared to the positive control group, which exhibited 100% pup mortality (Fig. 6, Table 4) with two out of 48 pups dying in the neonatal period, and the rest of pups succumbing to infection during the 27 days of postnatal observation. There were no significant differences between the treated groups, but statistical differences between treated groups and control groups were highly significant (*P* < 0.0001). In the group treated with ELQ-334, four out of 33 pups died in the neonatal period, followed by one death out of 29 in the postnatal period. Two out of six dams in this group presented also clinical signs for neosporosis. In the combined ELQ-334 + BKI-1748 group, eight out of 44 pups died in the neonatal period, followed by 100% survival until the end of the experiment. In this group also two dams out of seven presented a rough coat, indicative for limited clinical signs of neosporosis. In the group treated with ELQ-422, four out of 35 pups died in the neonatal period, followed by 100% survival during the postnatal phase. In this group none of the dams presented any clinical signs. However, in the group treated with ELQ-422 + BKI-1748, seven out of 39 pups died in the neonatal period followed by three pups succumbing to infection during the postnatal observation period. In the negative control group, one pup out of 19 died in the neonatal period, followed by 100% pup survival during the postnatal period until the end of the experiment.

**Table 4:** Effects of ELQs and BKI-1748 and their combination treatments on clinical signs, mortality, fertility, and cerebral NcSpain-7 infection in non-pregnant mice, dams, and pups. Mice were mated and infected with tachyzoites, and subsequently with ELQ-334, ELQ-422, BKI-1748, or respective combinations. Treatment of one group with corn oil alone was used as positive control. The negative control was kept uninfected but was treated with corn oil. Following euthanasia, adult mice and surviving pups were tested for the presence of N. caninum in the brain by quantitative real-time PCR. Pups that had died before the end of the experiment were considered N. caninum positive. Respective numbers of animals in the control and treated groups were compared between groups by the non-parametric Kruskal-Wallis test, followed by Mann-Whitney-U test or chi-square test.

	ELQ-334 5 mg/kg	ELQ-422 7.5 mg/kg	BKI-1748 20 mg/kg	ELQ-334 5 mg/kg + BKI-1748 20 mg/kg	ELQ-422 7.5 mg/kg + BKI-1748 20 mg/kg	Positive control	Negative control
NON-PREGNANT FEMALES							
**Total no.**	8/14	7/14	7/14	7/14	6/14	6/14	2/6
**Clinical signs**	0/8	0/7	0/7	0/7	0/6	0/6	0/2
***N. caninum* PCR positive**	0/8^a^	2/7^b^	4/7	1/7^c^	2/6	5/6	0/2
**DAMS**							
**Total no.**	6/14	7/14	7/14	7/14	8/14	8/14	4/6
**Clinical signs**	2/6	0/7	0/14	1/7	0/8	0/8	0/4
***N. caninum* PCR positive**	4/6	3/7^d^	5/7	1/7^e^	4/8^f^	8/8	0/4
**PUPS**							
**Litter size**	33	35	36	44	39	48	19
**Neonatal mortality**^i^	4/33	4/35	2/36	8/44	7/39	2/48	1/19
**Postnatal mortality**^j^	1/29^g^	0/31^g^	10/34^h^	0/36^g^	3/32^g^	46/46	0/18
***N. caninum* PCR positive**^k^	2/29	1/31	4/34	0/36	0/32	0/0	0/18

a *P* < 0.003.

b *P* < 0.0431.

c *P* < 0.0087.

d *P* < 0.0084.

e *P* < 0.0145.

f *P* < 0.0134.

g *P* < 0.0001.

h *P* < 0.0390.

i Proportion of pups born dead or that died within the 2 first days post-partum.

j proportion of pups that died during day 3–28 post-partum.

k PCR was performed with brains from surviving pups. Pups that died during the postnatal phase were regarded as PCR-positive (Dellarupe et al., 2014).

**Fig. 7:**
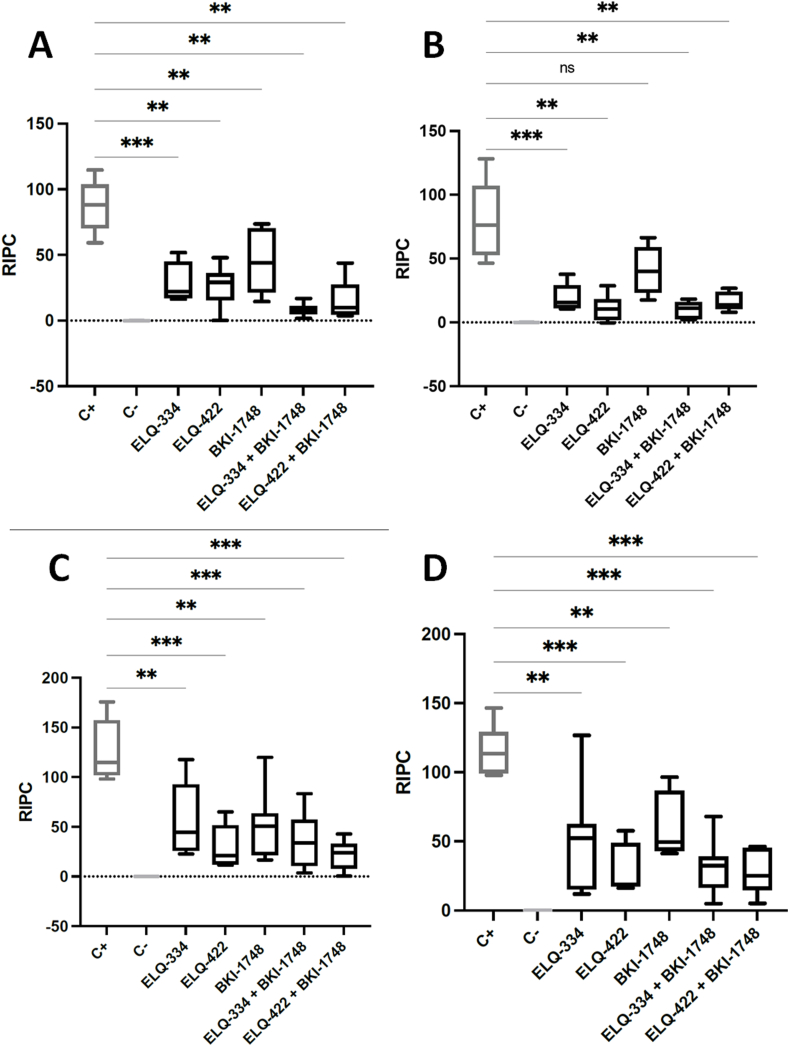
*N. caninum* IgG1 and IgG2a antibody titers measured from serum collected from non-pregnant mice and dams at the end of the experiment (42 days after challenge). Results are expressed as the mean of RIPC (relative index per cent) compared to the respective positive control (C+). (A) In non-pregnant mice, IgG1 levels between ELQ-334 and C+ were statistically significant (***P < 0.0007). Significant differences between IgG1 levels were also observed between ELQ-442 and C+ (***P < 0.0012). Significant differences in IgG1 antibody titers were observed between BKI-1748 treatment and the positive control (**P < 0.0082). IgG1 levels between the combined treatment (ELQ-334 + BKI-1748) and C+ were significantly different (**P < 0.0012) as well as the IgG1 levels of the combined treatment (ELQ-422 + BKI-1748) and C+ (**P < 0.0022). (B) In non-pregnant mice, IgG2a antibody titers between ELQ-334 and C+ were highly significant (***P < 0.0007). IgG2a levels between ELQ-422 compared to C+ were significantly different as well as the combination of ELQ-334 + BKI-1748 compared to the positive control (**P < 0.0012). Significant differences in IgG2a levels were also detected between the combination of ELQ-422 + BKI-1748 and C+ (**P < 0.0022). No significant IgG2a differences were measured between BKI-1748 and the positive control (ns = not significant). (C) In dams, IgG1 levels between ELQ-334 and C+ differs significantly (**P < 0.008). IgG1 antibody levels were highly significant between ELQ-422 and C+ (***P < 0.0003) but less significant between BKI-1748 and the positive control (**P < 0.0037). IgG1 levels between the combination of ELQ-334 + BKI-1748 and C+ were highly significant (***P < 0.0003) as well as the difference of IgG1 levels between the combination of ELQ-422 + BKI-1748 and the positive control (***P < 0.0002). (D) In dams, IgG2a titers of ELQ-334 compared to C+ was significantly different (**P < 0.0093). A highly significant difference of IgG2a levels was measured between ELQ-422 and C+ as well as between BKI-1748 and C+ (***P < 0.0007 and ***P < 0.0003). IgG2a levels between the combination of ELQ-334 + BKI-1748 and the positive control was also highly significant (***P < 0.0003) as well as the combination of ELQ-422 + BKI-1748 compared to C+ (***P < 0.0002). Antibody titres between treatment groups were compared by the non-parametric Kruskal-Wallis test, followed by Mann-Whitney-U test, included in the Graphpad Prism software.

Results on IgG1 and IgG2a levels in non-pregnant and pregnant mice are shown in Fig. 7. All infected mice displayed an antibody response against *N. caninum* antigens. However, in the treatment groups, antibody responses were significantly decreased compared to IgG1 and IgG2a levels in the sera of the positive control group. The only exception was found for IgG2a levels in BKI-1748 treated non-pregnant mice (Fig. 7 B).

**Fig. 8:**
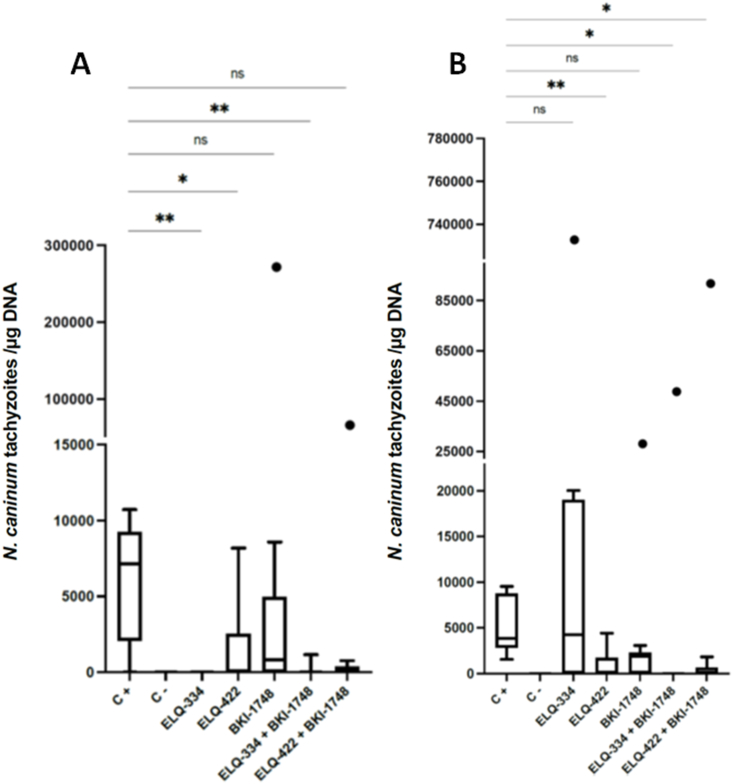
ELQ-334 (5 mg/kg), ELQ-422 (7.5 mg/kg)**,** BKI-1748 (20 mg/kg), as well as combined ELQ-334 (5 mg/kg) + BKI1748 (20 mg/kg) and ELQ-422 (7.5 mg/kg) + BKI-1748 (20 mg/kg) treatment in the pregnant neosporosis mouse model cerebral parasite burdens, shown in box plots. (A) In the non-pregnant mice, parasite burdens between ELQ-334 and C+ were statistically significant (**P < 0.0030), as well as between C+ and ELQ-422 (*P < 0.0431) and the combination between the combination of ELQ-334/BKI-1748 and the positive control (**P < 0.0087). Non-significant values (ns) were obtained when comparing the C+ with BKI-1748 as well as ELQ-422 combined with BKI-1748 compared to the positive control. (B) In the dams results, cerebral parasite burdens were highly statistically significant between the positive control and the group treated with ELQ-422 (**P < 0.0084). Statistical significance can be observed also between the C+ group and the two combination treatments, ELQ-334/BKI-1748 (*P < 0.0145) and the combination ELQ-422/BKI-1748 (*P < 0.0134). The comparisons between the positive control and ELQ-334 and BKI-1748 treated groups were not statistically significant (ns). Parasite burdens between treatment groups were compared by the non-parametric Kruskal-Wallis test, followed by Mann-Whitney-U test, included in the Graphpad Prism software.

The cerebral parasite load was assessed in pups and adult mice by quantitative real time PCR, and results are summarized in Table 4 and Fig. 8. Fig. 8 A displays the cerebral parasite burden in the non-pregnant mice. Without treatment, infection resulted in five out of six animals that tested *N. caninum* PCR-positive, while in the ELQ-334 treated group, a total eradication of the parasite load in the brain could be observed. In the ELQ-334 + BKI-1748 group, one out of seven mice was PCR-positive. In the group treated with ELQ-422, two out of seven mice were PCR-positive, and the same was found in the ELQ-422 + BKI-1748 group. Treatment with BKI-1748 alone resulted in four out of seven PCR-positive mice. Looking at the dams, all animals in the positive control group were PCR-positive (Table 4, Fig. 8 B). In the group treated with ELQ-334 only, *N. caninum* DNA was detected in four out of seven dams, while combining ELQ-334 with BKI-1748 reduced that number to one out of seven mice. Treatment with ELQ-422 resulted in three out of seven PCR-positive dams, compared to four out of eight in the combined treatment with BKI-1748. Application of BKI-1748 on its own resulted in three out of seven PCR-positive dams.

Vertical transmission of *N. caninum* to the pups was 100% in the positive control group, but clearly impaired upon drug treatments. ELQ-334 treatment reduced vertical transmission to 10.3%, ELQ-422 even more to 3.2%, and BKI-1748 treatment reduced vertical transmission to 41%. In the combination therapy groups, ELQ-422 + BKI-1748 resulted in a transmission rate of 9.4%, and vertical transmission was completely eradicated in the group treated with the ELQ-334 + BKI-1748 combination.

## 4 Discussion

The present study describes the efficacy of ELQ-316, its prodrugs ELQ-334 and ELQ-422, and of the bumped kinase inhibitor BKI-1748, either as individual treatments or as ELQ-BKI-combination treatments, *in vitro* and in a pregnant BALB/c mouse model for *N. caninum* infection.

Previous studies have already documented the *in vitro* activities of ELQ-316, its prodrug ELQ-334, and of BKI-1748 against Nc-β-gal tachyzoites (Anghel et al., 2018; Imhof et al., 2021). Here we show that ELQ-422 exhibited improved *in vitro* activity compared to ELQ-334 (EC_50_ = 15.6 μM compared to 54.2 μM, respectively). As ELQs target the mitochondrial electron transport chain and BKIs affect the activity of CDPK1, we hypothesized that combining these two drug classes would potentially increase the anti-parasitic activity and could have synergistic or additive effects.

*In vitro* Indeed, combining either ELQ-334 or ELQ-316 with BKI-1748 resulted in an EC_50_ below 0.1 nM, demonstrating that these compounds act in synergy. However, combining ELQ-422 and BKI-1748 resulted in a markedly higher EC_50_ of 53 nM, which was roughly three times higher than for ELQ-422 alone, but also about a third of the EC_50_ reported for BKI-1748. Why these differences in activity of the two combination treatments occur is not known. It is possible that drug-drug-interactions are involved (e.g. binding of ELQ-422 to BKI-1748), which would then reduce its activity, absorption and transport through membranes, or that ELQ-422 is less efficiently metabolized to ELQ-316 than ELQ-334 under these conditions. This needs to be further investigated.

We thus investigated how the combination of these two compounds would affect the morphology of the parasites *in vitro*. As observed previously for ELQ-316 and ELQ-334 (Anghel et al., 2018), treatments with ELQ-422 resulted in markedly slower *N. caninum* tachyzoite replication over time compared to non-treated controls. As evidenced by TEM, and in accordance with previous results obtained with ELQ-316 and ELQ-334, these parasites were largely viable after four days of treatment, retained their characteristic shape and size, remained located and underwent replication within a parasitophorous vacuole surrounded by a membrane, but exhibited distinct structural alterations mainly within the mitochondrial matrix. In contrast, and similar to other BKIs (Winzer et al, 2015, 2020a, 2020c), treatment with BKI-1748 resulted in the formation of multinucleated complexes containing newly formed zoites that were unable to separate and form novel tachyzoites. Combining ELQ-422 with BKI-1748 also lead to the formation of such intracellular complexes, albeit of much lower size and harbouring only few newly formed zoites. Thus, upon addition of the drugs to already infected HFF monolayers, an additive treatment effect could be observed.

MNC formation has also been described with other BKIs in *Neospora, Toxoplasma,* and *Besnoitia* (Jiménez-Meléndez et al., 2017; Winzer et al, 2015, 2020a). However, closer inspection of these smaller MNCs generated during the combination treatments revealed that they contained only few nuclei and exhibited an altered mitochondrial matrix, although these alterations appear less pronounced than with ELQ-422 alone. One explanation for this could be that mitochondrial damage is dependent on the proliferation rate, which is an energy dependent process. Since MNCs represent a relatively long-lived drug-induced stage of this parasite (Imhof et al., 2021; Winzer et al., 2020a) and a proteomics study has shown that the expression of a large portion of proteins that are involved in energy metabolism is downregulated (Winzer et al., 2020c), it is possible that in the combination treatment this will also impact the effects on the mitochondrion exerted by ELQ-422.

As ELQ-316 is characterized by low solubility *in vivo* (Doggett et al., 2020), animal studies were carried out with the prodrugs ELQ-334 and ELQ-422, which are metabolized to ELQ-316 rapidly, but increase solubility and thus compound efficacy (Miley et al., 2015). *N. caninum* infections have a strong impact in pregnancy outcome, and drug treatment against neosporosis is likely to take place during gestation. Thus, a promising compound should not interfere with fertility and successful delivery of healthy pups (Anghel et al., 2020). Our previous experiments had shown that ELQ-334 administered five times at 10 mg/kg/day to non-infected pregnant BALB/c did not have a strong interference in pregnancy (neonatal mortality in one out of 27 pups from four dams), while when the drug was applied to *N. caninum* infected pregnant mice, a considerable neonatal mortality (seven out of 44 pups from 10 dams) was observed (Anghel et al., 2018). In the current study, we found only low-level neonatal mortality induced by both ELQs applied at 10 mg/kg/day, and as shown previously, no effect of BKI-1748 treatment administered at 20 mg/kg (Imhof et al., 2021). However, combining these compounds resulted in an increase in neonatal mortality, especially for the ELQ-334 + BKI-1748 combination, raising a red flag indicating that especially that combination treatment could interfere in pregnancy outcome.

One reason for the higher neonatal mortality observed upon combination treatments could be drug-drug interactions, which can result in increased plasma levels (Sun et al., 2016). The comparison of plasma levels showed that especially in the ELQ-334 + BKI-1748 treatment group the drug levels were increased for both compounds compared to the individual compound treatments. For BKI-1748, treatments at 50 mg/kg/day for five days have been reported to cause foetal loss in the mouse model, while 20 mg/kg/day did not interfere in pregnancy. Thus, increased plasma levels in the ELQ-334 + BKI-1748 combination treatment could explain partial adverse effects in pregnant mice (Anghel et al., 2018; Imhof et al., 2021). In contrast, the combined ELQ-422 + BKI-1748 treatment did not lead to increased plasma levels for these compounds, and this mirrors the only moderate neonatal mortality and lack of postnatal mortality in the pregnancy interference test. Taking in account the literature, where it is stated that combinations of treatments can increase plasma levels, and possibly require lower dosages of compounds which act synergistically but with a higher effect (Sun et al., 2016), we adjusted the dosage for the subsequent challenge experiment: ELQ-334 was applied at 5 mg/kg/day, and ELQ-442 was applied at 7.5 mg/kg/day, while the BKI-1748 dosage of 20 mg/kg/day was maintained.

The treatments of *N. caninum*-infected BALB/c mice during days 9–13 of pregnancy with ELQ-334 and ELQ-422 at lower dosages resulted in improved fertility rates and overall number of new-born pups. All infected mice were serologically positive for *N. caninum*, and the efficacy of the drug treatments was mirrored by decreased IgG1 and IgG2a levels in the drug treated groups.

In non-pregnant mice, treatment with ELQ-334 at 5 mg/kg/day was highly efficient and completely prevented brain infection as determined by real time PCR. This is surprising, since in a previous study the cerebral parasite burden in non-pregnant mice treated with 10 mg/kg of ELQ-334 was not notably reduced compared to the placebo group (Anghel et al., 2018). This indicates that the higher dosage could have detrimental rather than beneficial effects, and for this compound the amount used for treatment could potentially be even more reduced without losing the anti-parasitic effect. In all other experimental treatment groups, including the combination treatments, infection of the brain of non-pregnant mice was not completely inhibited, but no clinical signs were noted.

In the dams, mild clinical signs occurred in two out of six mice in the ELQ-334 and one out of six mice in the ELQ-334 + BKI-1748 group, but not in the other groups. The ELQ-334 group also exhibited a high cerebral parasite burden while the combination with BKI-1748 led to a marked reduction of cerebral parasite load. The two combination treatments resulted in very low cerebral parasite load in the dams and in the non-pregnant mice, and no clinical signs were noted. Despite higher parasite load and a higher number of animals with *Neospora* positive brains, no clinical signs were noted in the BKI-1748 treated group. The fact that BKI-1748 does not have a notable impact on CNS infection in dams has been shown previously. The distinct differences between non-pregnant mice and dams in terms of cerebral parasite load can be explained by the immunomodulation towards a Th2 biased response that takes place during pregnancy (Aguado-Martínez et al., 2017).

In terms of neonatal mortality of pups, no significant differences were noted between all groups, but postnatal mortality was clearly reduced in the drug treated groups compared to the positive control, where all animals succumbed to infection and were considered *Neospora* positive (Dellarupe et al., 2014). Two groups, namely the group receiving ELQ-422 and the ELQ-334 + BKI-1748 combination treatment, exhibited 100% postnatal survival. The vertical transmission was also reduced in all groups compared to the control, but the most notable effect was observed in the group treated with ELQ-334 + BKI-1748, where vertical transmission of *N. caninum* to the pups was completely inhibited.

For an optimal treatment, there is always a trade-off between neonatal and postnatal mortality and vertical transmission. Overall, the combination treatments are a valuable option as opposed to single drug applications. However, in the case of neosporosis the situation is special as these combination treatments are applied during pregnancy. Thus, it will be important in further studies to investigate the adjustment of the dosages, taking into account the results presented here: while we had shown previously that ELQ-334 administered at 10 mg/kg/day led to a 50% reduced vertical transmission (Anghel et al., 2018), we here show that a lower dose of 5 mg/kg/day inhibits pup infection by > 90%, and in combination with BKI-1748 at 20 mg/kg, vertical transmission is completely blocked. It is noteworthy to mention that a combination of ELQ-334 and another cytochrome *bc1* inhibitor, atovaquone, resulted in complete cure of experimental *Babesia microti* infection in immunodeficient mice (Lawres et al., 2016), and treatments of persistently *T. gondii* infected mice with ELQ-334 resulted in a dramatic reduction of tissue cyst in the brain and the combination of ELQ-334 with pyrimethamine further reduces the number of brain cysts (Doggett et al., 2020; Martynowicz et al., 2020).

In conclusion, our results show that the combination between ELQ-316 and its two prodrugs, ELQ-334 and ELQ-422, and BKI-1748 which have different targets and are singly very potent compounds against *N. caninum in vitro* and *in vivo,* can increase the plasma levels of both compounds when applied in combination, and can reduce vertical transmission in experimentally infected mice. Also, by applying a combined treatment, the doses of the compounds could be reduced, with the same or increased effect on the vertical transmission.

## Author contributions

NA, DI and AH conceived and designed the study. AH coordinated the biological assays. NA, DI, PW, JR, KH and VB carried out *in vitro* and *in vivo* experimental work. RC, MAH, GRW, SLMA, KKO, WCVV and JSD provided the compound and produced PK data, LMOM provided the Nc-Spain7 isolate. NA and DI carried out statistical analysis. WCVV, KKO, LMOM, AH, NA and DI did interpretation of results. NA, DI and AH wrote the manuscript. All authors corrected and approved the manuscript.

## Funding

This study was financed by the 10.13039/100000001Swiss National Science Foundation (SNSF) grant 310030_184662, the 10.13039/100000002National Institutes of Health (NIH) grants R01AI089441, R01AI111341, R01HD080670, R01AI155412, R01HD102487, R21AI123690, and R21AI140881, and 10.13039/100000199United States Department of Agriculture, 10.13039/100005825National Institute of Food and Agriculture grants # 2019-07512 and # 2014–06183, and the 10.13039/100000738U.S. Department of Veterans Affairs Biomedical Laboratory Research and Development Career Development Award BX002440 and VA Merit Review Award BX004522.

## Declaration of competing interest

WCVV is an owner/officer of ParaTheraTech Inc, a company which is seeking to bring bumped kinase inhibitors to the animal health market.

All other authors have no competing interests.

## References

[bib1] Aguado-Martínez A., Basto A.P., Leitão A., Hemphill A. (2017). Neospora caninum in non-pregnant and pregnant mouse models: cross-talk between infection and immunity. Int. J. Parasitol..

[bib2] Aguado-Martinez A., Basto A.P., Müller J., Balmer V., Manser V., Leitaõ A., Hemphill A. (2016). N-terminal fusion of a toll-like receptor 2-ligand to a Neospora caninum chimeric antigen efficiently modifies the properties of the specific immune response. Parasitology.

[bib3] Aguado-Martínez A., Basto A.P., Tanaka S., Ryser L.T., Nunes T.P., Ortega-Mora L.M., Arranz-Solís D., Leitão A., Hemphill A. (2019). Immunization with a cocktail of antigens fused with OprI reduces Neospora caninum vertical transmission and postnatal mortality in mice. Vaccine.

[bib4] Alaeddine F., Hemphill A., Debache K., Guionaud C. (2013). Molecular cloning and characterization of NcROP2Fam-1, a member of the ROP2 family of rhoptry proteins in Neospora caninum that is targeted by antibodies neutralizing host cell invasion in vitro. Parasitology.

[bib5] Anghel N., Balmer V., Müller J., Winzer P., Aguado-Martinez A., Roozbehani M., Pou S., Nilsen A., Riscoe M., Doggett J.S., Hemphill A. (2018). Endochin-like quinolones exhibit promising efficacy against neospora caninum in vitro and in experimentally infected pregnant mice. Front. Vet. Sci..

[bib6] Anghel N., Winzer P.A., Imhof D., Müller J., Langa X., Rieder J., Barrett L.K., Vidadala R.S.R., Huang W., Choi R., Hulverson M.A., Whitman G.R., Arnold S.L., Van Voorhis W.C., Ojo K.K., Maly D.J., Fan E., Hemphill A. (2020). Comparative assessment of the effects of bumped kinase inhibitors on early zebrafish embryo development and pregnancy in mice. Int. J. Antimicrob. Agents.

[bib7] Arranz-Solís D., Aguado-Martínez A., Müller J., Regidor-Cerrillo J., Ortega-Mora L.M., Hemphill A. (2015). Dose-dependent effects of experimental infection with the virulent Neospora caninum Nc-Spain7 isolate in a pregnant mouse model. Vet. Parasitol..

[bib8] Barna F., Debache K., Vock C.A., Küster T., Hemphill A. (2013). In Vitro effects of novel ruthenium complexes in Neospora caninum and Toxoplasma gondii tachyzoites. Antimicrob. Agents Chemother..

[bib9] Björkman C., Hemphill A. (1998). Characterization of Neospora caninum iscom antigens using monoclonal antibodies. Parasite Immunol..

[bib10] Castellanos-Gonzalez A., White A.C., Ojo K.K., Vidadala R.S.R., Zhang Z., Reid M.C., Fox A.M.W., Keyloun K.R., Rivas K., Irani A., Dann S.M., Fan E., Maly D.J., Van Voorhis W.C. (2013). A novel calcium-dependent protein kinase inhibitor as a lead compound for treating cryptosporidiosis. J. Infect. Dis..

[bib11] Debache K., Alaeddine F., Guionaud C., Monney T., Müller J., Strohbusch M., Leib S.L., Grandgirard D., Hemphill A. (2009). Vaccination with recombinant NcROP2 combined with recombinant NcMIC1 and NcMIC3 reduces cerebral infection and vertical transmission in mice experimentally infected with Neospora caninum tachyzoites. Int. J. Parasitol..

[bib12] Debache K., Guionaud C., Alaeddine F., Mevissen M., Hemphill A. (2008). Vaccination of mice with recombinant NcROP2 antigen reduces mortality and cerebral infection in mice infected with Neospora caninum tachyzoites. Int. J. Parasitol..

[bib13] Dellarupe A., Regidor-Cerrillo J., Jiménez-Ruiz E., Schares G., Unzaga J.M., Venturini M.C., Ortega-Mora L.M. (2014). Clinical outcome and vertical transmission variability among canine Neospora caninum isolates in a pregnant mouse model of infection. Parasitology.

[bib14] Doggett J.S., Nilsen A., Forquer I., Wegmann K.W., Jones-Brando L., Yolken R.H., Bordón C., Charman S.A., Katneni K., Schultz T., Burrows J.N., Hinrichs D.J., Meunier B., Carruthers V.B., Riscoe M.K. (2012). Endochin-like quinolones are highly efficacious against acute and latent experimental toxoplasmosis. Proc. Natl. Acad. Sci. U.S.A..

[bib15] Doggett J.S., Ojo K.K., Fan E., Maly D.J., Van Voorhis W.C. (2014). Bumped kinase inhibitor 1294 treats established toxoplasma gondii infection. Antimicrob. Agents Chemother..

[bib16] Doggett J.S., Schultz T., Miller A.J., Bruzual I., Pou S., Winter R., Dodean R., Zakharov L.N., Nilsen A., Riscoe M.K., Carruthers V.B. (2020). Orally bioavailable endochin-like quinolone carbonate ester prodrug reduces toxoplasma gondii brain cysts. Antimicrob. Agents Chemother..

[bib17] Dubey J.P., Carpenter J.L., Speer C.A., Topper M.J., Uggla A. (1988). Newly recognized fatal protozoan disease of dogs. J. Am. Vet. Med. Assoc..

[bib18] Dubey J.P., Hemphill A., Calero-Bernal R., Rafael), Schares G. (2017).

[bib19] Dubey J.P., Jenkins M.C., Rajendran C., Miska K., Ferreira L.R., Martins J., Kwok O.C.H., Choudhary S. (2011). Gray wolf (Canis lupus) is a natural definitive host for Neospora caninum. Vet. Parasitol..

[bib20] Gondim L.F.P., McAllister M.M., Pitt W.C., Zemlicka D.E. (2004). Coyotes (Canis latrans) are definitive hosts of Neospora caninum. Int. J. Parasitol..

[bib21] Guionaud C., Hemphill A., Mevissen M., Alaeddine F. (2010). Molecular characterization of Neospora caninum MAG1, a dense granule protein secreted into the parasitophorous vacuole, and associated with the cyst wall and the cyst matrix. Parasitology.

[bib22] Horcajo P., Regidor-Cerrillo J., Aguado-Martínez A., Hemphill A., Ortega-Mora L.M. (2016). Vaccines for bovine neosporosis: current status and key aspects for development. Parasite Immunol..

[bib23] Hulverson M.A., Bruzual I., McConnell E.V., Huang W., Vidadala R.S.R., Choi R., Arnold S.L.M., Whitman G.R., McCloskey M.C., Barrett L.K., Rivas K.L., Scheele S., DeRocher A.E., Parsons M., Ojo K.K., Maly D.J., Fan E., Van Voorhis W.C., Doggett J.S. (2019). Pharmacokinetics and in vivo efficacy of pyrazolopyrimidine, pyrrolopyrimidine, and 5-aminopyrazole-4-carboxamide bumped kinase inhibitors against toxoplasmosis. J. Infect. Dis..

[bib24] Imhof D., Anghel N., Winzer P., Balmer V., Ramseier J., Choi R., Hulverson M.A., Whitman G.R., Samuel L., Arnold M., Ojo K.K., Voorhis W.C. Van, Doggett J.S., Ortega-mora L.M., Hemphill A. (2021). In vitro activity, safety and in vivo efficacy of the novel bumped kinase inhibitor BKI-1748 in non-pregnant and pregnant mice experimentally infected with Neospora caninum tachyzoites and Toxoplasma gondii oocysts. Int. J. Parasitol. Drugs Drug Resist..

[bib25] Jiménez-Meléndez A., Ojo K.K., Wallace A.M., Smith T.R., Hemphill A., Balmer V., Regidor-Cerrillo J., Ortega-Mora L.M., Hehl A.B., Fan E., Maly D.J., Van Voorhis W.C., Álvarez-García G. (2017). In vitro efficacy of bumped kinase inhibitors against Besnoitia besnoiti tachyzoites. Int. J. Parasitol..

[bib26] Kappagoda S., Singh U., Blackburn B.G. (2011). Antiparasitic therapy. Mayo Clin. Proc..

[bib27] Keyloun K.R., Reid M.C., Choi R., Song Y., Fox A.M.W., Hillesland H.K., Zhang Z., Vidadala R., Merritt E.A., Lau A.O.T., Maly D.J., Fan E., Barrett L.K., Van Voorhis W.C., Ojo K.K. (2014). Parasitology.

[bib28] Kieschnick H., Wakefield T., Narducci C.A., Beckers C. (2001). Toxoplasma gondii attachment to host cells is regulated by a calmodulin-like domain protein kinase. J. Biol. Chem..

[bib29] King J.S., Šlapeta J., Jenkins D.J., Al-Qassab S.E., Ellis J.T., Windsor P.A. (2010). Australian dingoes are definitive hosts of Neospora caninum. Int. J. Parasitol..

[bib30] Lawres L.A., Garg A., Kumar V., Bruzual I., Forquer I.P., Renard I., Virji A.Z., Boulard P., Rodriguez E.X., Allen A.J., Pou S., Wegmann K.W., Winter R.W., Nilsen A., Mao J., Preston D.A., Belperron A.A., Bockenstedt L.K., Hinrichs D.J., Riscoe M.K., Doggett J.S., Mamoun C. Ben (2016).

[bib31] Martynowicz J., Doggett J.S., Sullivan W.J. (2020 Aug 20). Efficacy of guanabenz combination therapy against chronic toxoplasmosis across multiple mouse strains. Antimicrob. Agents Chemother..

[bib32] McAllister M.M., Dubey J.P., Lindsay D.S., Jolley W.R., Wills R.A., McGuire A.M. (1998). Dogs are definitive hosts of Neospora caninum. Int. J. Parasitol..

[bib33] McCann C.M., Vyse A.J., Salmon R.L., Thomas D., Williams D.J.L., McGarry J.W., Pebody R., Trees A.J. (2008). Lack of serologic evidence of neospora caninum in humans, england. Emerg. Infect. Dis..

[bib34] McConnell E.V., Bruzual I., Pou S., Winter R., Dodean R.A., Smilkstein M.J., Krollenbrock A., Nilsen A., Zakharov L.N., Riscoe M.K., Doggett J.S. (2018). Targeted structure-activity analysis of endochin-like quinolones reveals potent Qi and qo site inhibitors of toxoplasma gondii and Plasmodium falciparum cytochrome bc1 and identifies ELQ-400 as a remarkably effective compound against acute experimental to. ACS Infect. Dis..

[bib35] Miley G.P., Pou S., Winter R., Nilsen A., Li Y., Kelly J.X., Stickles A.M., Mather M.W., Forquer I.P., Pershing A.M., White K., Shackleford D., Saunders J., Chen G., Ting L.M., Kim K., Zakharov L.N., Donini C., Burrows J.N., Vaidya A.B., Charman S.A., Riscoe M.K. (2015). ELQ-300 prodrugs for enhanced delivery and single-dose cure of malaria. Antimicrob. Agents Chemother..

[bib36] Monney T., Hemphill A. (2014). Vaccines against neosporosis: what can we learn from the past studies?. Exp. Parasitol..

[bib37] Müller J., Aguado-Martínez A., Balmer V., Maly D.J., Fan E., Ortega-Mora L.M., Ojo K.K., Van Voorhis W.C., Hemphill A. (2017). Two novel calcium-dependent protein kinase 1 inhibitors interfere with vertical transmission in mice infected with Neospora caninum tachyzoites. Antimicrob. Agents Chemother..

[bib38] Müller J., Aguado-Martínez A., Manser V., Wong H.N., Haynes R.K., Hemphill A. (2016). Repurposing of antiparasitic drugs: the hydroxy-naphthoquinone buparvaquone inhibits vertical transmission in the pregnant neosporosis mouse model. Vet. Res..

[bib39] Müller J., Aguado-Martínez A., Ortega-Mora L.M., Moreno-Gonzalo J., Ferre I., Hulverson M.A., Choi R., McCloskey M.C., Barrett L.K., Maly D.J., Ojo K.K., Voorhis W. Van, Hemphill A. (2017). Development of a murine vertical transmission model for Toxoplasma gondii oocyst infection and studies on the efficacy of bumped kinase inhibitor (BKI)-1294 and the naphthoquinone buparvaquone against congenital toxoplasmosis. J. Antimicrob. Chemother..

[bib40] Müller N., Vonlaufen N., Gianinazzi C., Leib S.L., Hemphill A. (2002). Application of real-time fluorescent PCR for quantitative assessment of Neospora caninum infections in organotypic slice cultures of rat central nervous system tissue. J. Clin. Microbiol..

[bib41] Nilsen A., LaCrue A.N., White K.L., Forquer I.P., Cross R.M., Marfurt J., Mather M.W., Delves M.J., Shackleford D.M., Saenz F.E., Morrisey J.M., Steuten J., Mutka T., Li Y., Wirjanata G., Ryan E., Duffy S., Kelly J.X., Sebayang B.F., Zeeman A.M., Noviyanti R., Sinden R.E., Kocken C.H.M., Price R.N., Avery V.M., Angulo-Barturen I., Jiménez-Díaz M.B., Ferrer S., Herreros E., Sanz L.M., Gamo F.J., Bathurst I., Burrows J.N., Siegl P., Guy R.K., Winter R.W., Vaidya A.B., Charman S.A., Kyle D.E., Manetsch R., Riscoe M.K. (2013). Quinolone-3-diarylethers: a new class of antimalarial drug. Sci. Transl. Med..

[bib42] Ojo K.K., Dangoudoubiyam S., Verma S.K., Scheele S., DeRocher A.E., Yeargan M., Choi R., Smith T.R., Rivas K.L., Hulverson M.A., Barrett L.K., Fan E., Maly D.J., Parsons M., Dubey J.P., Howe D.K., Van Voorhis W.C. (2016). Selective inhibition of Sarcocystis neurona calcium-dependent protein kinase 1 for equine protozoal myeloencephalitis therapy. Int. J. Parasitol..

[bib43] Ojo K.K., Reid M.C., Siddaramaiah L.K., Müller J., Winzer P., Zhang Z., Keyloun K.R., Vidadala R.S.R., Merritt E.A., Hol W.G.J., Maly D.J., Fan E., Van Voorhis W.C., Hemphill A. (2014). Neospora caninum calcium-dependent protein kinase 1 is an effective drug target for neosporosis therapy. PloS One.

[bib44] Pedroni M.J., Vidadala R.S.R., Choi R., Keyloun K.R., Reid M.C., Murphy R.C., Barrett L.K., Van Voorhis W.C., Maly D.J., Ojo K.K., Lau A.O.T. (2016). Bumped kinase inhibitor prohibits egression in Babesia bovis. Vet. Parasitol..

[bib45] Pink R., Hudson A., Mouriès M.A., Bendig M. (2005). Opportunities and challenges in antiparasitic drug discovery. Nat. Rev. Drug Discov..

[bib46] Reichel M.P., Alejandra Ayanegui-Alcérreca M., Gondim L.F.P., Ellis J.T. (2013). What is the global economic impact of Neospora caninum in cattle - the billion dollar question. Int. J. Parasitol..

[bib47] Rosenthal P.J. (2003). Antimalarial drug discovery: old and new approaches. J. Exp. Biol..

[bib48] Rutaganira F.U., Barks J., Dhason M.S., Wang Q., Lopez M.S., Long S., Radke J.B., Jones N.G., Maddirala A.R., Janetka J.W., El Bakkouri M., Hui R., Shokat K.M., Sibley L.D. (2017). Inhibition of calcium dependent protein kinase 1 (CDPK1) by pyrazolopyrimidine analogs decreases establishment and reoccurrence of central nervous system disease by toxoplasma gondii. J. Med. Chem..

[bib49] Sánchez-Sánchez R., Ferre I., Re M., Ramos J.J., Regidor-Cerrillo J., Díaz M.P., González-Huecas M., Tabanera E., Benavides J., Hemphill A., Hulverson M.A., Barrett L.K., Choi R., Whitman G.R., Ojo K.K., Van Voorhis W.C., Ortega-Mora L.M. (2019). Treatment with bumped kinase inhibitor 1294 is safe and leads to significant protection against abortion and vertical transmission in sheep experimentally infected with toxoplasma gondii during pregnancy. Antimicrob. Agents Chemother..

[bib50] Sánchez-Sánchez R., Ferre I., Re M., Vázquez P., Ferrer L.M., Blanco-Murcia J., Regidor-Cerrillo J., Pizarro Díaz M., González-Huecas M., Tabanera E., García-Lunar P., Benavides J., Castaño P., Hemphill A., Hulverson M.A., Whitman G.R., Rivas K.L., Choi R., Ojo K.K., Barrett L.K., Van Voorhis W.C., Ortega-Mora L.M. (2018). Safety and efficacy of the bumped kinase inhibitor BKI-1553 in pregnant sheep experimentally infected with Neospora caninum tachyzoites. Int. J. Parasitol. Drugs Drug Resist..

[bib51] Schorer M., Debache K., Barna F., Monney T., Müller J., Boykin D.W., Stephens C.E., Hemphill A. (2012). Di-cationic arylimidamides act against Neospora caninum tachyzoites by interference in membrane structure and nucleolar integrity and are active against challenge infection in mice. Int. J. Parasitol. Drugs Drug Resist..

[bib52] Shrestha A., Ojo K.K., Koston F., Ruttkowski B., Vidadala R.S.R., Dorr C.S., Navaluna E.D., Whitman G.R., Barrett K.F., Barrett L.K., Hulverson M.A., Choi R., Michaels S.A., Maly D.J., Hemphill A., Van Voorhis W.C., Joachim A. (2019). Bumped kinase inhibitor 1369 is effective against Cystoisospora suis in vivo and in vitro. Int. J. Parasitol. Drugs Drug Resist..

[bib53] Stickles A.M., Smilkstein M.J., Morrisey J.M., Li Y., Forquer I.P., Kelly J.X., Pou S., Winter R.W., Nilsen A., Vaidya A.B., Riscoe M.K. (2016). Atovaquone and ELQ-300 combination therapy as a novel dual-site cytochrome bc1 inhibition strategy for malaria. Antimicrob. Agents Chemother..

[bib54] Stickles A.M., Ting L.M., Morrisey J.M., Li Y., Mather M.W., Meermeier E., Pershing A.M., Forquer I.P., Miley G.P., Pou S., Winter R.W., Hinrichs D.J., Kelly J.X., Kim K., Vaidya A.B., Riscoe M.K., Nilsen A. (2015). Inhibition of cytochrome bc1 as a strategy for single-dose, multi-stage antimalarial therapy. Am. J. Trop. Med. Hyg..

[bib55] Sun W., Sanderson P., Zheng W. (2016). Drug combination therapy increases successful drug repositioning. Drug Discov. Today.

[bib56] Van Voorhis W.C., Doggett J.S., Parsons M., Hulverson M.A., Choi R., Arnold S.L.M., Riggs M.W., Hemphill A., Howe D.K., Mealey R.H., Lau A.O.T., Merritt E.A., Maly D.J., Fan E., Ojo K.K. (2017). Extended-spectrum antiprotozoal bumped kinase inhibitors: a review. Exp. Parasitol..

[bib57] Whitten M.K. (1957). Effect of exteroceptive factors on the œstrous cycle of mice [40]. Nature.

[bib58] Winter R., Kelly J.X., Smilkstein M.J., Hinrichs D., Koop D.R., Riscoe M.K. (2011). Optimization of endochin-like quinolones for antimalarial activity. Exp. Parasitol..

[bib59] Winzer P., Anghel N., Imhof D., Balmer V., Ortega-Mora L.M., Ojo K.K., van Voorhis W.C., Müller J., Hemphill A. (2020). Neospora Caninum: Structure and Fate of Multinucleated Complexes Induced by the Bumped Kinase Inhibitor Bki-1294. Pathogens.

[bib60] Winzer P., Imhof D., Anghel N., Ritler D., Müller J., Boubaker G., Aguado-Martinez A., Ortega-Mora L.M., Ojo K.K., VanVoorhis W.C., Hemphill A. (2020). The impact of BKI-1294 therapy in mice infected with the apicomplexan parasite neospora caninum and Re-infected during pregnancy. Front. Vet. Sci..

[bib61] Winzer P., Müller J., Aguado-Martínez A., Rahman M., Balmer V., Manser V., Ortega-Mora L.M., Ojo K.K., Fan E., Maly D.J., Van Voorhis W.C., Hemphill A. (2015). In vitro and in vivo effects of the bumped kinase inhibitor 1294 in the related cyst-forming apicomplexans Toxoplasma gondii and Neospora caninum. Antimicrob. Agents Chemother..

[bib62] Winzer P., Müller J., Imhof D., Ritler D., Uldry A.-C., Braga-Lagache S., Heller M., Ojo K.K., Van Voorhis W.C., Ortega-Mora L.-M., Hemphill A. (2020). Neospora caninum: differential proteome of multinucleated complexes induced by the bumped kinase inhibitor BKI-1294. Microorganisms.

